# Method of Shortening the Period of Pain during the Action of Arsenical Applications

**Published:** 1898-05

**Authors:** 


ARTICLE II.

### METHOD OF SHORTENING THE PERIOD OF PAIN DURING THE ACTION OF ARSENICAL APPLICATIONS.

The action of arsenious acid on the dentin and the pulp, and a method to shorten the pain during its time of application, was recently discussed in a paper read by Dr. Rjabkoff before the International Medical Congress, section of Odontology.

Says he, in the *Oesterreichisch-Ungarische Vierteljahrsschrift:*

"The pain which caused the application of arsenious acid on pulps in a certain state of inflammation can often become very perseving. The same has been attributed to the caustic action of the acid, which one tried to alleviate by the addition of some astringents and anesthetics. After long experimentation with pastes of different compositions, it was found that those admixtures did not improve the action of the acid; on the contrary, the same had become uncertain and weak. The distributing influence of these admixtures can be proven in the following: Cocain causes the pulp to dry up and become anemic. Now, if it is admitted that it is absorbed by the pulp before or together with the acid, the above must be the consequence. The action of the acid is then held back, because it is a fact that devitalization takes quicker steps in pulps rich in blood."

Again, the powdery admixtures, as morphine and tannin, will always be on the surface of the paste, owing to their specific lightness. Only a little of the acid comes in contact with the pulp, therefore Dr. Rjabkoff tried to find out other means to alleviate and shorten the period of pain. Relying on the fact that arsenious acid causes painless devitalization or sound, entirely exposed pulps, he came to the conclusion that in cases of pulp it is the presence of an intervening layer of tissue that must prevent necessarily the penetration of the acid deep into the pulp. The acid, coming in contact with the layer, will be absorbed by same; a scurf will be formed, which depends entirely on the thickness and structure of the inflamed tissue, as also on the pureness and consistency of the paste.

Should now, as it occasionally does, the scurfy layer of tissue rupture and the mass of paste get below it, in direct contact with a larger surface of the pulp, then a quick and painless devitaliz ıtion takes place.

Only in these cases the pastes with admixtures have given satisfaction, and they have led to their general use.

The removal of the intervening layer of inflamed tissue would cause the acid to act much quicker. But as it is in most cases utterly useless to even try to remove it directly, on account of intense pain, Dr. Rjabkoff succeeded in the following way: He applied a good paste for ten to twenty minutes to the inflamed tissue; with a probe he then destroyed this layer, which could be done with very little pain, because it was perfectly devitalized. He then brought more paste on the probe in direct contact with the pulp mass, and found that the pain was not as intense any more and ceased entirely after the cavity had been closed up air-tight. The pain usually agonizing patients for hours had been reduced ten to twenty minutes, which the writer claimed a big success.—*F. A. B., in Dental Weekly.*

